# Application of anterior minimally invasive clamping technique combined with lower extremity axial bone traction device in irreducible intertrochanteric fractures

**DOI:** 10.3389/fsurg.2025.1584651

**Published:** 2025-09-03

**Authors:** Yunliang Zhu, Baoliang Lu, Changwu Ouyang, Sichao Gu

**Affiliations:** ^1^ Department of Orthopedics, Gaoyou People's Hospital, The Third Clinical Medical College of Yangzhou University, Yangzhou, Jiangsu, China; ^2^ Department of Orthopedics, The First Affiliated Hospital of USTC, Division of Life Sciences and Medicine, University of Science and Technology of China, Hefei, Anhui, China; ^3^ The Second Affiliated Hospital of Fuyang Normal University, Fuyang, Anhui, China; ^4^ Department of Orthopaedic, Coal Mine Construction General Hospital, Suzhou, Anhui, China

**Keywords:** intertrochanteric fracture, irreducible, clamping technique, lower extremity axial bone traction device, hip joint function

## Abstract

**Objective:**

This study aims to evaluate the effectiveness of the anterior minimally invasive clamping technique in conjunction with a lower extremity axial bone traction device for treating irreducible intertrochanteric fractures.

**Methods:**

We conducted a retrospective analysis of data from 69 patients with irreducible intertrochanteric fractures who underwent limited open reduction and intramedullary nail fixation at our hospital between January 2022 and October 2023. All patients had subtrochanteric fractures of the femur. Patients received treatment using the anterior minimally invasive clamping technique combined with a lower extremity axial bone traction device for reduction (clamping + bone traction device group). The cohort included 27 males and 42 females with a mean age of (71.32 ± 5.11) years (range, 60–83 years). Sixty-nine patients with irreducible intertrochanteric fractures were matched for gender and age in a 1:1 ratio. The 1:1 matching process was performed using a propensity score matching method to ensure comparability between the two groups. Key matching variables included comorbidities (hypertension, diabetes mellitus, coronary heart disease, and cerebrovascular disease), with a maximum allowable difference of one comorbidity between matched pairs. Treatment schedules were strictly aligned, meaning patients in both groups received surgery within 72 h of admission, and preoperative management (including anti-coagulation and pain control) followed the same protocol. For fracture patterns, the AO/OTA classification was used as a critical matching criterion: each patient in the clamping + bone traction device group was matched with a patient in the clamping + traction bed group with the same AO/OTA subtype (31-A1, 31-A2, or 31-A3). The matching tolerance for fracture displacement (assessed by preoperative x-ray) was set at <2 mm to ensure similar fracture severity. A caliper width of 0.2 standard deviations of the propensity score was used to minimize selection bias, and the balance of baseline characteristics after matching was verified using standardized mean differences (all <0.1, indicating good balance). The control group (clamping + traction bed group) consisted of patients treated during the same period using limited open reduction and intramedullary nailing with an anterior minimally invasive clamping technique combined with a traction bed. This group included 30 males and 39 females with a mean age of (69.49 ± 6.59) years (range, 54–86 years). We compared the two groups regarding surgical indicators, postoperative recovery, and the quality of fracture reduction. The Harris functional score was used to assess hip joint function at baseline, as well as 6 and 12 months post-surgery. Record and compare the levels of mMPTA and mLDFA between two groups before surgery, 1 month after surgery, and 3 months after surgery. We also recorded the occurrence of postoperative complications in both groups.

**Results:**

In the clamping + bone traction device group, the operation time, intraoperative blood loss, and number of fluoroscopic images were (78.49 ± 15.29) minutes, (242.25 ± 15.65) ml, and (15.52 ± 3.12) times, respectively. These values were significantly lower than those in the clamping + traction bed group, which were (85.57 ± 12.18) minutes, (251.20 ± 19.45) ml, and (17.14 ± 2.95) times (*P* < 0.05). The length of hospital stay, time to assist in ambulation, and fracture healing time for the clamping + bone traction device group were 13.00 (12.00, 13.00) days, (15.84 ± 3.10) hours, and (15.38 ± 2.35) weeks, respectively, which were shorter compared to the clamping + traction bed group: 15.00 (13.00, 16.00) days, (19.75 ± 4.28) hours, and (16.77 ± 2.41) weeks, with significant differences (*P* < 0.05). The quality of fracture reduction was better in the clamping + bone traction device group than in the clamping + traction bed group, with significant differences (*P* < 0.05). The Harris functional scores for the clamping + bone traction device group were (53.29 ± 3.08), (60.84 ± 5.06), (72.33 ± 4.21), and (88.29 ± 6.78) at 1, 3, 6, and 12 months post-surgery, respectively. These scores were higher than those of the clamping + traction bed group, which were (50.86 ± 4.18), (56.23 ± 4.24), (68.52 ± 3.46), and (85.33 ± 5.56) (*P* < 0.05). The mMPTA, mLDFA levels for the clamping + bone traction device group were (87.63 ± 4.41)°, (90.82 ± 5.53)°, (88.92 ± 7.44)°, (91.62 ± 7.73)°at 1, 3 months post-surgery, respectively. These scores were higher than those of the clamping + traction bed group, which were (85.55 ± 5.57)°, (88.40 ± 4.12)°, (85.51 ± 8.05)°, (88.34 ± 7.25)° (*P* < 0.05). The incidence of postoperative complications in the clamping + bone traction device group was 4.35%, significantly lower than the 14.49% in the clamping + traction bed group (*P* < 0.05).

**Conclusion:**

The anterior minimally invasive clamping technique combined with a lower limb axial bone distraction device in patients with irreducible intertrochanteric fractures can reduce operation time, minimize intraoperative blood loss and fluoroscopy usage, enhance fracture reduction quality, lower the occurrence of postoperative complications, and promote fracture healing and recovery of hip joint function.

## 1 Introduction

Intertrochanteric fractures represent about 1.4% of all fractures in the body and are more prevalent among middle-aged and elderly individuals, resulting in high rates of disability and mortality (1). As the global population ages, the incidence of intertrochanteric fractures has risen each year. It is projected that by 2050, the number of affected individuals will increase from 1.66 million in 1990 to 6.26 million. This trend will significantly impact patient health and quality of life while also placing immense strain on healthcare systems worldwide. Intertrochanteric fractures have become a pressing public health concern (2). Currently, the preferred treatment for intertrochanteric fractures is closed reduction. However, this method does not always achieve satisfactory anatomical alignment and often necessitates larger surgical incisions, tools, and other supportive techniques. These challenging fractures require minimally invasive, efficient, and effective reduction, which is a key focus in treating irreducible intertrochanteric fractures (3). Achieving a satisfactory reduction of irreducible intertrochanteric fractures in a minimally invasive, quick, and effective manner is the primary focus of clinical treatment. This challenge is one of the key issues that needs to be addressed urgently.

Limited open reduction and intramedullary nailing are common approaches for irreducible intertrochanteric fractures. During surgery, traction beds are typically used to facilitate reduction; however, the design of these beds can obstruct intraoperative fluoroscopy, and a certain angle between the traction gravity line and the mechanical axis of the lower limb may hinder effective fracture reduction (4). Moreover, the unique anatomical features of the intertrochanteric region, along with the tension from the gluteus medius, gluteus minimus, iliopsoas, and various ligaments, make it challenging for patients to maintain fracture alignment. This can compromise intraoperative imaging and other reduction efforts, potentially lowering the quality of fracture reduction and elevating the risk of complications such as nonunion, malalignment, and impaired limb function (5).

Recently, researchers have explored alternatives to traction beds for assisting with reduction and ensuring stability. Among these, the lower extremity axial bone traction device and the anterior minimally invasive clamping technique are frequently utilized. The support point of the lower extremity axial bone traction device is aligned with the body's force line structure, allowing for a rapid restoration of lower limb alignment and providing ample space for surgery to proceed smoothly. The anterior minimally invasive clamping technique facilitates further correction of fracture alignment through a smaller incision while effectively stabilizing the fracture site, promoting the orderly execution of subsequent reduction procedures and achieving favorable outcomes (6, 7).

Traditionally, the lower extremity axial bone traction device and the anterior minimally invasive clamping technique have been employed independently in fracture reduction surgeries (8, 9). However, few studies have examined the potential synergistic effects of combining these two methods for treating irreducible intertrochanteric fractures. This study aims to retrospectively analyze the data of patients with irreducible intertrochanteric fractures who underwent limited open reduction and intramedullary nail fixation. We will compare the outcomes of patients treated using the anterior minimally invasive clamping technique with the lower extremity axial bone traction device against those treated with the anterior minimally invasive clamping technique combined with a traction bed. The study objectives include: (1) comparing surgical indicators between the two groups; (2) analyzing differences in reduction outcomes, postoperative recovery, and hip joint function, highlighting the benefits of the anterior minimally invasive clamping technique with the lower extremity axial bone traction device; and (3) exploring the safety and feasibility of combining these reduction techniques.

## 2 Data and methods

### 2.1 Study subjects

Irreducible intertrochanteric fractures (10): intertrochanteric fractures that could not achieve satisfactory reduction through closed manipulation after three or more attempts, characterized by persistent displacement of fracture fragments (including varus/valgus deformity >10°, anterior/posterior tilt >10°, or lateral displacement >5 mm) confirmed by intraoperative fluoroscopy. Satisfactory reduction was defined as fracture displacement <2 mm, with varus, valgus, anteversion, or posterior tilt angle <5°.

Inclusion criteria: (1) Patients with intertrochanteric fractures that meet the diagnostic criteria outlined in the *Surgery* (10), all of which are unilateral fractures; (2) Patients for whom closed reduction failed to achieve satisfactory results after three attempts; (3) Patients who underwent initial limited open reduction and intramedullary nail fixation treatment with successful outcomes; (4) Patients with a postoperative follow-up period of at least 12 months; (5) Patients with complete data.

Exclusion criteria: (1) Patients with fractures in other locations; (2) Patients with severe multiple injuries or uncontrolled underlying systemic diseases; (3) Patients with pathological fractures; (4) Patients with open fractures; (5) Patients with lower limb disabilities or functional impairments before the fracture; (6) Patients with a history of surgical trauma to the lower limb on the fractured side.

### 2.2 General data

We conducted a retrospective analysis of the clinical data from 69 patients with irreducible intertrochanteric fractures treated with limited open reduction and intramedullary nail fixation at our hospital between January 2022 and October 2023. All patients had subtrochanteric fractures of the femur. These patients were treated using the anterior minimally invasive clamping technique combined with a lower extremity axial bone traction device (referred to as the clamping + bone traction device group). The cohort included 27 males and 42 females, with a mean age of (71.32 ± 5.11) years (range: 60–83 years).

We matched this sample by age and gender in a 1:1 ratio with another group of 69 patients who received similar treatment during the same period, but used the anterior minimally invasive clamping technique combined with a traction bed (referred to as the clamping + traction bed group). This second group consisted of 30 males and 39 females, with a mean age of (69.49 ± 6.59) years (range: 54–86 years). Statistical analysis showed no significant differences in the general data between the two groups (*P* > 0.05, Table 1).

**Table 1:** Comparison of general data of patients in the clamping + bone traction device group and the clamping + traction bed group.

Group	Number of cases	Age ($\bar{x}$±*s*, years)	Gender (male/female, cases)	AO classification of fracture (31-A1/31-A2/31-A3, cases)	Cause of fracture (fall/traffic accident/other)	Fracture site (left/right)
Clamping + bone traction device	69	71.32 ± 5.11	27/42	16/32/21	9/53/7	28/41
Clamping + traction bed	69	69.49 ± 6.59	30/39	12/35/22	12/51/6	25/44
Statistical value	–	*t* = 1.819	*χ*^2^ = 0.269	*χ*^2^ = 0.729	*χ*^2^ = 0.544	*χ*^2^ = 0.276
*P*	–	0.071	0.604	0.695	0.762	0.600

This study was reviewed and approved by the Medical Ethics Committee of the hospital.

The AO/OTA classification system was used to categorize the intertrochanteric fractures in this study. Type 31-A1 fractures are simple trochanteric fractures with a stable medial cortex; 31-A2 fractures are multifragmentary trochanteric fractures with an unstable medial cortex but intact posteromedial support; 31-A3 fractures are intertrochanteric fractures extending into the subtrochanteric region with complete loss of posteromedial support. All fracture classifications were independently confirmed by two senior orthopedic surgeons with more than 10 years of experience, and any discrepancies were resolved through consensus.

### 2.3 Surgical methods

After the administration of spinal or general anesthesia, patients in the clamping + bone traction device group were positioned supine on a fluoroscopic operating table. Following routine disinfection and draping, a Steinmann nail was inserted from the anterior inferior iliac spine to the bone of the greater sciatic notch, with the tail of the needle tilted 15°–20° toward the distal end of the affected limb. A second Steinmann nail was then inserted from the femoral malleolus to the medial malleolus. A sleeve was attached to the proximal Steinmann nail, and an extension rod was connected in the middle. The proximal and distal ends were linked to a complete set of lower limb axial bone traction device components. A C-arm x-ray machine (Guangdong Smartop Medical Technology Co., Ltd., China; Approval No. 20202060168, Model: RG-III-MDR) was employed to assess the overall alignment and displacement of the fracture ends. Manual traction reduction was initially performed; once the extension rod was locked to maintain length, the distal component of the traction device was rotated for further traction reduction. After achieving anatomical reduction, a small longitudinal incision (1.0–1.5 cm) was made in the anterior and lateral or inferior lateral area of the greater trochanter to expose the fracture ends. A point reduction clamp was used to secure, correct, and stabilize the fracture site through the incision. Once the fracture was adequately reduced under fluoroscopy, a longer incision (approximately 5 cm) was made from the apex of the femoral trochanter to the proximal end. The fascia and muscle layers were bluntly separated with a vascular clamp to reveal the fracture ends. A guide needle was positioned slightly inside the apex of the greater trochanter, and after expanding the medullary cavity, an anti-rotation intramedullary nail was inserted. With the aid of a sight, a guide needle and a spiral blade were sequentially inserted into the femoral neck, followed by a locking nail at the distal end. After reconfirming satisfactory fracture reduction using fluoroscopy, the equipment was removed, and the wound was rinsed and sutured layer by layer before applying a dressing.

**Figure 1:**
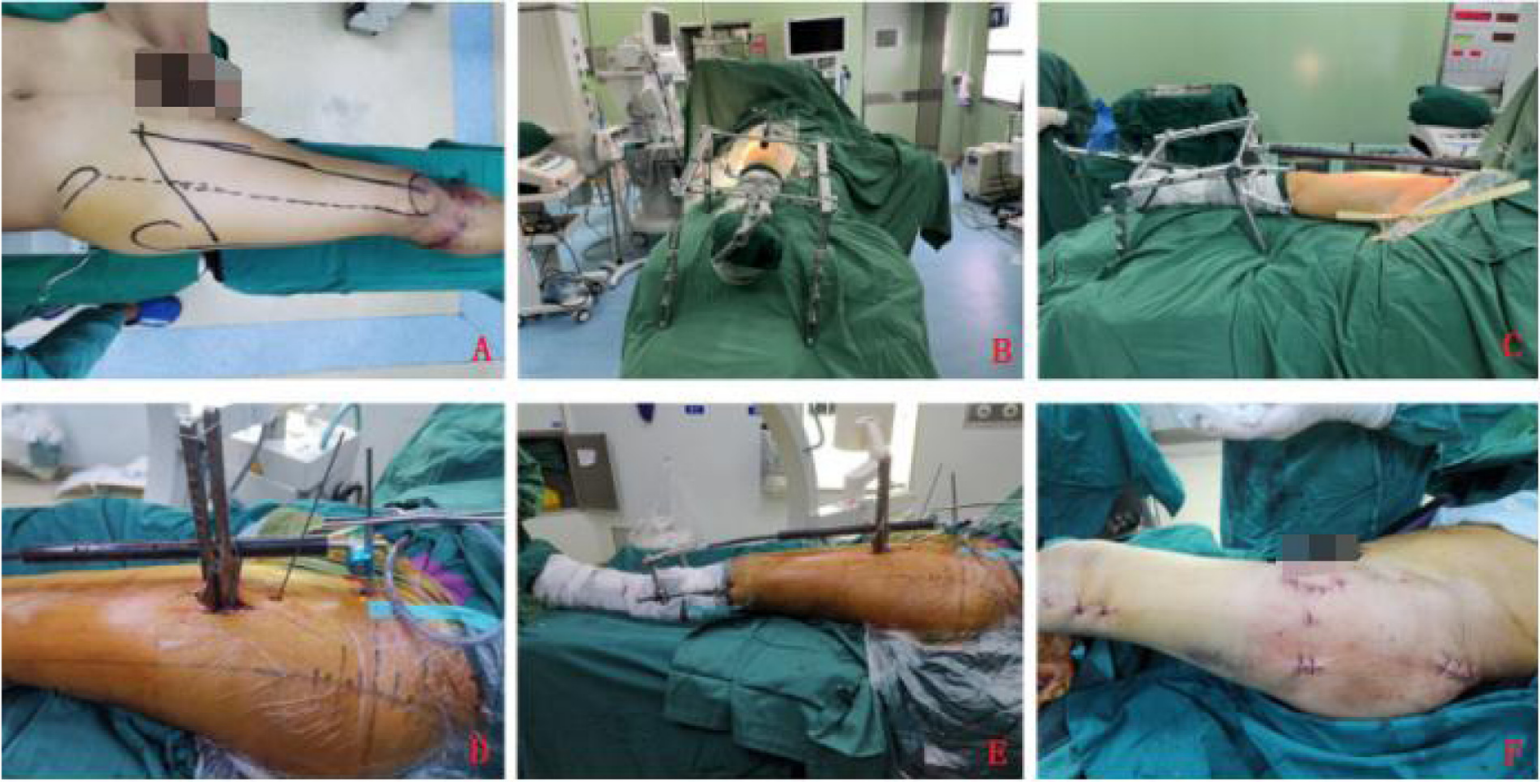
**(A)** Shows preoperative planning; **(B–E)** depict intraoperative procedures; **(F)** shows postoperative incision presentation.

For the clamping + traction bed group, after either intraspinal or general anesthesia, the patient was placed supine on the assembled traction bed with the foot of the affected limb secured in a traction boot. The C-arm x-ray machine was used to observe the overall force line and the displacement of the fracture ends. Based on these observations, longitudinal traction reduction was performed using the traction bed. After confirming a good reduction, the clamp maintenance and subsequent intramedullary nail placement were conducted in the same manner as in the clamping + bone traction device group (Figure 1).

### 2.4 Postoperative treatment

Patients received symptomatic treatment post-surgery, including anti-infection measures, pain relief, and anticoagulation. Within 24 h after surgery, rehabilitation activities began under the guidance of a rehabilitation doctor. These activities included passive or active flexion and extension exercises of the hip joint. One to two days post-surgery, patients gradually started to get out of bed using a walker, with the duration of activity increasing according to their tolerance.

### 2.5 Follow-up and observation indicators

All patients were followed up in the outpatient clinic at 1, 3, 6, and 12 months after surgery to assess fracture healing.

Surgical indicators for both groups were recorded, including the total length of the incision, operation time, blood loss during surgery, and number of intraoperative fluoroscopy events.

Postoperative recovery metrics were also documented, such as length of hospital stay, time to mobilization with an assistive device, and time to fracture healing.

X-rays were taken at the first follow-up examination 1 month after surgery, and the quality of fracture reduction was evaluated according to the criteria established by Kasha et al. (11). The grading was as follows: Excellent: fracture displacement <2 mm, varus, valgus, anteversion, or posterior tilt angle <5°. Good: fracture displacement 2–5 mm, varus, valgus, anteversion, or retroversion angle 5°–10°; Poor: fracture displacement >5 mm, with corresponding angles >5°.

The Harris Functional Scoring Method (12) was used to evaluate hip joint function for both groups before surgery and at 1, 3, 6, and 12 months after surgery. The scale measures pain (0–44 points), function (0–47 points), deformity (0–4 points), and range of motion (0–5 points), with a total possible score of 100 points; higher scores indicate better hip joint function. The Cronbach's α for this scale was 0.826.

Preoperative and postoperative 1 and 3 months, take full-length x-rays of the lower limbs, measure the proximal medial tibial mechanical angle (mMPTA), distal lateral femoral mechanical angle (mLDFA), and knee joint range of motion (ROM), all 3 times, and take the mean.

Postoperative complications for both groups were recorded, including issues such as loss of reduction, nonunion, perineal injury, and deep vein thrombosis of the lower extremities.

### 2.6 Statistical analysis

Statistical analysis was performed using SPSS version 25.0. Measurement data underwent the Shapiro–Wilk normality test. Data meeting normal distribution were expressed as mean ± standard deviation. The independent sample *t*-test was used for inter-group comparisons, the paired sample *t*-test for intra-group comparisons, and the repeated measures test for data collected at multiple time points. Skewed data were expressed as [M (P25, P75)], with the Mann–Whitney *U* test used for inter-group comparisons. Categorical data were presented as frequency (cases, %) and analyzed using the *χ*^2^ test. A statistical significance level (α) of 0.05 was set for two-sided tests. For all continuous variables, the Shapiro–Wilk test confirmed that data conforming to normal distribution (including operation time, intraoperative blood loss, number of intraoperative fluoroscopy, Harris functional scores, mMPTA, and mLDFA) passed the normality test (*P* > 0.05). For group comparisons of normally distributed data, effect sizes were calculated using Cohen's d, with values of 0.2, 0.5, and 0.8 indicating small, medium, and large effects, respectively. Specifically, Cohen's d values for operation time, intraoperative blood loss, and number of intraoperative fluoroscopy were 0.49, 0.48, and 0.53, respectively, suggesting medium effects. Regarding multiple comparisons, the Bonferroni correction was applied to adjust for type I errors when analyzing repeated measures data (e.g., Harris scores and mMPTA/mLDFA at different time points), ensuring the overall significance level remained at *α* = 0.05.

## 3 Results

### 3.1 Surgery-related indicators

The operation time for the clamping + bone traction device group was shorter than that for the clamping + traction bed group. Additionally, intraoperative blood loss and fluoroscopy times were less for the clamping + bone traction device group, with statistical significance (*P* < 0.05, Table 2). There was no statistically significant difference in the total length of the incision between the two groups (*P* > 0.05, Table 2).

**Table 2:** Comparison of surgical indicators between the clamping + bone traction device group and the clamping + traction bed group ($\bar{x}$±s).

Group	Number of cases	Total length of incision (cm)	Operation time (min)	Intraoperative blood loss (ml)	Number of intraoperative fluoroscopy (times)
Clamping + bone traction device	69	6.82 ± 0.89	78.49 ± 15.29	242.25 ± 15.65	15.52 ± 3.12
Clamping + traction bed	69	7.05 ± 1.01	85.57 ± 12.18	251.20 ± 19.45	17.14 ± 2.95
*t*	–	1.368	3.006	2.980	3.138
*P* value	–	0.174	0.003	0.003	0.002

### 3.2 Postoperative recovery

The hospitalization time, time to assisted ambulation, and time to fracture healing were shorter in the clamping + bone traction device group compared to the clamping + traction bed group, all showing statistically significant differences (*P* < 0.05, Table 3).

**Table 3:** Comparison of postoperative recovery between the clamping + bone traction device group and the clamping + traction Bed group ($\bar{x}$**±*s***).

Group	Number of cases	Hospitalization time (d)	Time to get out of bed with assistive device (h)	Fracture healing time (weeks)
Clamping + bone traction device	69	13.00 (12.00, 13.00)	15.84 ± 3.10	15.38 ± 2.35
Clamping + traction bed	69	15.00 (13.00, 16.00)	19.75 ± 4.28	16.77 ± 2.41
Statistical value	–	*Z* = 2.798	*t* = 6.152	*t* = 3.433
*P* value	–	0.005	<0.001	<0.001

### 3.3 Fracture reduction quality

The quality of fracture reduction was superior in the clamping + bone traction device group, with a statistically significant difference (*P* < 0.05, Table 4).

**Table 4:** Comparison of fracture reduction quality between the clamping + bone traction device group and the clamping + traction bed group *n* (%).

Group	Number of cases	Excellent	Good	Poor
Clamping + bone traction device	69	48 (69.57)	19 (27.54)	2 (2.90)
Clamping + traction bed	69	37 (53.62)	25 (36.23)	7 (10.14)
*Z*	–	2.093		
*P* value	–	0.036		

### 3.4 Hip joint function (Harris function score)

Before surgery, there was no statistically significant difference in the Harris function scores between the two groups (*P* > 0.05, Table 5). However, at 1, 3, 6, and 12 months after surgery, the Harris function scores for both groups increased, with scores for the clamping + bone traction device group consistently higher at each time point, reflecting statistically significant differences (*P* < 0.05, Table 5).

**Table 5:** Comparison of Hip joint function between the clamping + bone distractor group and the clamping + traction Bed group ($\bar{x}$**±**s, points).

Group	Number of cases	Preoperative	1 month after surgery	3 months after surgery	6 months after surgery	12 months after surgery
Clamping + bone traction device	69	44.80 ± 2.98	53.29 ± 3.08^a^^,^ ^b^	60.84 ± 5.06^a^^,^ ^b^^,^ ^c^	72.33 ± 4.21^a^^,^ ^b^^,^ ^c^^,^ ^d^	88.29 ± 6.78^a^^,^ ^b^^,^ ^c^^,^ ^d^^, e^
Clamping + traction bed	69	45.88 ± 4.04	50.86 ± 4.18^b^	56.23 ± 4.24^b^^,^ ^c^	68.52 ± 3.46^b^^,^ ^c^^,^ ^d^	85.33 ± 5.56^b^^,^ ^c^^,^ ^d^^, e^
*F*_Inter-group_ *P*	–	49.984/<0.001				
*F*_Time Point_ *P*	–	1,877.175/<0.001				
*F*_interaction between groups and time points_ *P*	–	8.415/<0.001				

Comparisons with the clamp + traction bed group at the same time point indicate statistically significant differences.

^a^ *P* < 0.05, vs. the same group before surgery.

^b^ *P* < 0.05, vs. the same group 1 month after surgery.

^c^ *P* < 0.05, vs. the same group 3 months after surgery.

^d^ *P* < 0.05, vs. the same group 6 months after surgery.

^e^ *P* < 0.05, vs. the same group 6 months after surgery.

### 3.5 Postoperative complications

The incidence of postoperative complications in the clamping + bone traction device group was lower than in the clamping + traction bed group, and this difference was statistically significant (*P* < 0.05, Table 6).

**Table 6:** Comparison of postoperative complications between the clamping + bone traction device group and the clamping + traction bed group *n* (%).

Group	Number of cases	Loss of reduction	Nonunion	Perineal injury	Deep vein thrombosis of lower limbs	Total incidence
Clamping + bone traction device	69	0	0	0	3 (4.35)	3 (4.35)
Clamping + traction bed	69	3 (4.35)	1 (1.45)	1 (1.45)	5 (7.25)	10 (14.49)
*χ* ^2^	–	–	–	–	–	4.161
*P* value	–	–	–	–	–	0.041

### 3.6 mMPTA, mLDFA

Before surgery, there was no statistically significant difference in the mMPTA, mLDFA levels between the two groups (*P* > 0.05, Table 7). However, at 1, 3 months after surgery, the mMPTA, mLDFA levels for both groups increased, with scores for the clamping + bone traction device group consistently higher at each time point, reflecting statistically significant differences (*P* < 0.05, Table 7).

**Table 7:** Comparison of mMPTA, mLDFA between the clamping + bone traction device group and the clamping + traction bed group **(**$\bar{x}$**±s**).

Group	Number of cases	mMPTA (°)			mLDFA (°)		
		Preoperative	1 month after surgery	3 months after surgery	Preoperative	1 month after surgery	3 months after surgery
Clamping + bone traction device	69	84.33 ± 5.51	87.63 ± 4.41^a^^,^ ^b^	90.82 ± 5.53^a^^,^ ^b^^,^ ^c^	83.93 ± 6.21	88.92 ± 7.44^a^^,^ ^b^	91.62 ± 7.73^a^^,^ ^b^^,^ ^c^
Clamping + traction bed	69	83.79 ± 6.34	85.55 ± 5.57^a^^,^ ^b^	88.40 ± 4.12^a^^,^ ^b^^,^ ^c^	83.62 ± 6.58	85.51 ± 8.05^a^^,^ ^b^	88.34 ± 7.25^a^^,^ ^b^^,^ ^c^
*F*_Inter-group_ *P*	–	31.635/<0.001					
*F*_Time Point_ *P*	–	126.871/<0.001					
*F*_interaction between groups and time points_ *P*	–	9.362/<0.001					

Comparisons with the clamp + traction bed group at the same time point indicate statistically significant differences.

^a^ *P* < 0.05, vs. the same group before surgery.

^b^ *P* < 0.05, vs. the same group 1 month after surgery.

^c^ *P* < 0.05, vs. the same group 3 months after surgery.

### 3.7 Typical case

**Figure 2:**
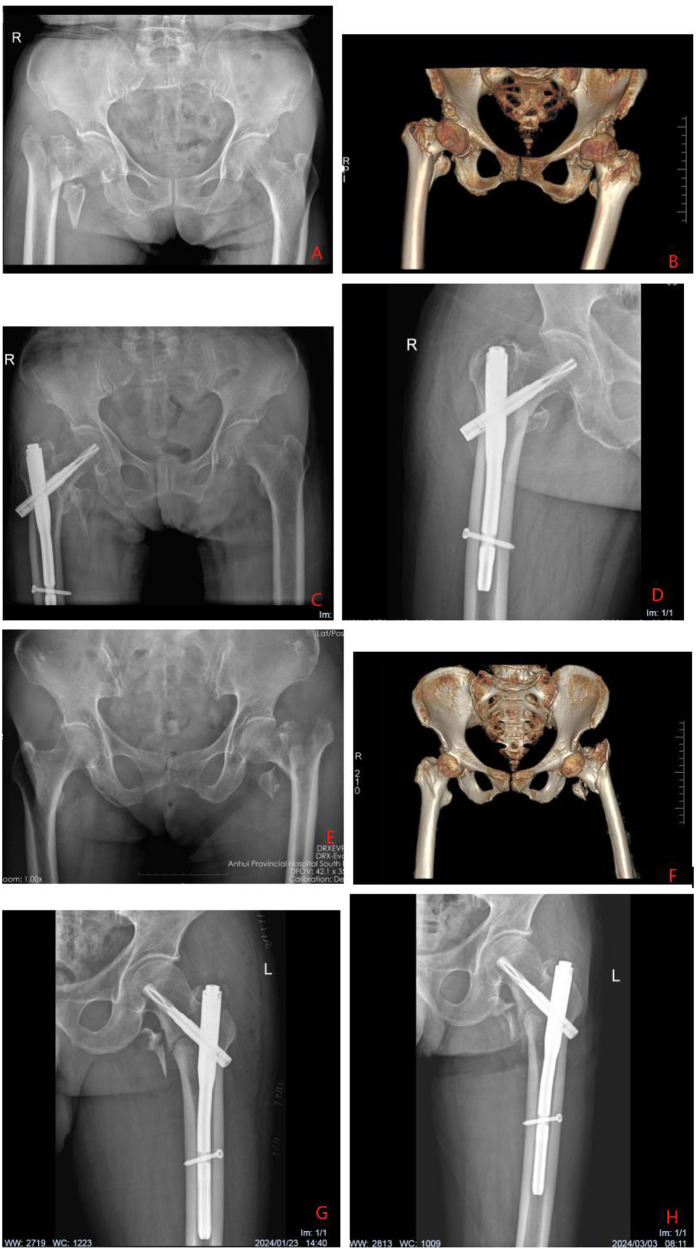
**(A–D)** Shows a 75 year old male patient with right intertrochanteric fracture. According to ao/ota classification, the fracture is 31a2. Who was treated with clamping and bone traction device. **(A)** Shows the preoperative anteroposterior radiograph of both hips (1:15 cm), **(B)** shows the preoperative three-dimensional reconstruction radiograph of both hips (1:20 cm), **(C)** shows the anteroposterior radiograph of both hips (1:15 cm) on the first day after operation, and **(D)** shows the anteroposterior radiograph of the right hip (1:10 cm) on the third month after operation. **(E–H)** Shows a 75 year old male patient with left intertrochanteric fracture. The ao/ota classification is 31a2. Who was treated with clamping and traction bed. **(A)** Shows the preoperative anteroposterior radiograph of both hips (1:15 cm), **(B)** shows the preoperative three-dimensional reconstruction radiograph of both hips (1:20 cm), **(C)** shows the anteroposterior radiograph of the left hip on the day after operation (1:10 cm), and **(D)** shows the anteroposterior radiograph of the left hip on the third month after operation (1:10 cm).

Figures 2A–D show preoperative and postoperative images of Clamping + Bone Tractio patients; Figures 2E–H show preoperative and postoperative images of patients with Clamping + Traction Bed. It can be seen that the postoperative recovery of Clamping + Bone Tractio patients is better than that of Clamping + Traction Bed patients (Figure 2).

## 4 Discussion

The proximal femur is part of the metaphyseal transition zone. On the outer side of the upper end of the femur is the greater trochanter, while the inner side features the lesser trochanter. Both the greater trochanter, lesser trochanter, and the intertrochanter are composed of cancellous bone. Serving as the junction between the femoral neck and femoral shaft, the femoral trochanters experience the highest shear stress (13). When a fracture occurs between the trochanters, the greater trochanter becomes prominent, and there is no soft tissue to cushion the area. Furthermore, surrounding muscle groups and soft tissues exert pulling forces, leading to diverse fracture morphologies and end displacement modes in intertrochanteric fractures. Conventional closed reduction often fails to achieve an optimal result, potentially leading to an irreducible intertrochanteric fracture (14). Patients with these types of fractures typically require limited incisions and traction reduction under direct visualization to ensure better outcomes. This study retrospectively analyzed the efficacy of the anterior minimally invasive clamping technique combined with a lower limb axial bone traction device, as well as the same technique combined with a traction bed for reducing irreducible intertrochanteric fractures. The results indicated that the combination of the anterior minimally invasive clamping technique and lower extremity axial bone traction device was simpler, quicker, provided better reduction, and resulted in fewer postoperative complications compared to using the traction bed. This approach positively impacted fracture healing and hip joint function recovery in patients.

### 4.1 Early clinical efficacy of anterior minimally invasive clamping technique combined with lower extremity axial bone traction device reduction in patients with irreducible intertrochanteric fractures

The findings of this study revealed that patients using the bone traction device experienced shorter operation times, less blood loss, and fewer fluoroscopy instances compared to those using the traction bed. Patients with the bone traction device also had shorter durations of assisted ambulation, faster fracture healing, shorter hospital stays, and better quality of fracture reduction, reflected in higher postoperative Harris functional scores. This improvement may be attributed to the fact that the traction bed primarily provides skin traction, resulting in a relatively small force and limited reduction efficacy (15). Zhang et al. (16) noted that the traction bed's complex structure requires multiple people for assembly and can obstruct the surgical field, affecting the reduction process and fluoroscopy results. This complexity can lead to increased fluoroscopy times, prolonged operation durations, higher blood loss, and decreased reduction effectiveness. In contrast, the lower limb axial bone traction device has a straightforward design, requiring only two Steinmann wires for installation. This allows the surgeon to quickly set it up without assistance, providing ample space for surgical maneuvers and enabling greater freedom of movement for the patient's lower limbs. If necessary, the device allows adjustments to the patient's position, facilitating a smoother surgical process, which shortens operation times and minimizes blood loss (17). Additionally, the mechanical principle of the lower extremity axial bone traction device aligns with the “homeopathic reduction” concept proposed by Academician Zhang Yingze. By supporting the distal end of the femoral neck and the femoral condyle of the affected limb, the traction device applies a more powerful force directly to the bone compared to the skin traction of the traction bed. The symmetrical tension generated by the axial traction helps align the fracture fragments more effectively, promoting a quicker recovery of the lower limb's force line, expediting the surgical process, reducing the need for repeated fluoroscopy, and ensuring effective reduction (18). Enhanced reduction outcomes facilitate earlier postoperative ambulation, accelerate fracture healing, and support the recovery of hip joint function. Long et al. (19) compared the effects of a bone traction device with a traction bed-assisted femoral neck dynamic cross nail system for reducing and fixing femoral neck fractures. They found that using the bone traction device simplified the reduction process, reduced the time required, and led to fewer intraoperative fluoroscopy sessions, resulting in better early recovery of hip joint function. Their findings suggest that, compared to traction bed reduction, the lower extremity axial bone traction device offers superior outcomes in patients with irreducible intertrochanteric fractures.

Additionally, a previous study has indicated that proper maintenance after the reduction of irreducible intertrochanteric fractures is critical for the surgical process and overall reduction success (20). However, in Long et al.'s study, only bone traction devices were employed to reduce the fracture site, without utilizing other methods to assist in reduction and stabilization. In this study, we introduced a minimally invasive approach that used a small incision to correct and maintain the fracture site after reduction with the lower extremity axial bone traction device. This was achieved through a clamping technique involving a reduction clamp, which allowed for correction of multi-plane displacements, including in the sagittal and coronal planes. This technique helps restore the physiological alignment of the bone fragments, correct lateral cortical displacement, and achieve complete closed reduction of the fracture site. The bolts on the reduction clamp also effectively stabilize the fracture site, preventing re-displacement due to manual maintenance and reducing the need for repeated fluoroscopy and bleeding caused by multiple reductions. Ultimately, this approach shortens operation time, decreases intraoperative fluoroscopy rates, and minimizes blood loss (21). Furthermore, effective maintenance of fracture reduction via the anterior minimally invasive clamping technique establishes a solid foundation for subsequent intramedullary nail fixation. This contributes to better internal fixation, enhances the quality of fracture reduction, prevents postoperative malunion, and aids in the recovery of the hip joint's anatomical structure and function (8). Zhao et al. (22) also reported that in treating patients with irreducible intertrochanteric fractures using limited open reduction and intramedullary nail fixation, the fracture site was clamped and reduced through a small incision in the pelvic cavity. This technique achieved minimally invasive reduction, decreased the complexity of the procedure, and reduced trauma, ultimately improving fracture reduction outcomes and facilitating post-surgery recovery of hip function. The combined use of anterior minimally invasive clamping technology and the lower extremity axial bone traction device can yield better effects in fracture reduction and stabilization, promote rapid postoperative recovery, and enhance hip joint function.

### 4.2 Effect of anterior minimally invasive clamping technology combined with lower extremity axial bone traction device reduction on postoperative complications

The results of this study indicated that the incidence of postoperative complications in the clamping + bone traction device group was lower than in the clamping + traction bed group. This suggests that using anterior minimally invasive clamping technology alongside a lower extremity axial bone traction device is beneficial in reducing the risk of postoperative complications for patients with irreducible intertrochanteric fractures. One possible explanation is that when traditional traction beds are employed to manage these fractures, insufficient traction force can hinder effective reduction, increasing the risk of postoperative issues such as loss of reduction and nonunion. Conversely, excessive traction force may cause damage to the perineum due to over-compression, leading to genital edema and perineal nerve paralysis, which can hinder early postoperative mobility and ultimately raise the risk of lower extremity deep vein thrombosis (23). The lower extremity axial bone traction device has a straightforward structure, and the bilateral traction lines align with the mechanical axis of the lower extremity. During the reduction process, it can effectively work with the soft tissue structures surrounding the fracture, minimizing complications associated with unilateral traction or insufficient force. This combination achieves effective reduction while avoiding damage to perineal tissues and nerves, allowing patients to mobilize sooner after surgery and reducing the risk of deep vein thrombosis from prolonged bed rest (24). Furthermore, the combined use of anterior minimally invasive clamping technology and the lower limb axial bone traction device enhances the maintenance of fracture reduction, facilitates effective fixation with intramedullary nails, improves overall reduction outcomes, and decreases the likelihood of postoperative complications, including loss of reduction and nonunion (25).

### 4.3 Limitations

This study has several limitations: (1) As a single-center retrospective non-randomized study, it is inherently subject to selection bias. Patient allocation to treatment groups depended on clinical decisions rather than randomization, and despite efforts to balance baseline characteristics using propensity score matching, residual bias cannot be fully excluded. The limited sample size further may compromise statistical power, highlighting the need for multicenter studies with larger cohorts to validate the findings. (2) The patient matching and subgroup analysis have limitations. There is a lack of comparative analysis on reduction effectiveness across different patient types, and although key variables (comorbidities, treatment schedules, and AO/OTA classification) were matched, unmeasured factors related to fracture complexity or patient-specific characteristics might still affect outcomes. (3) Issues related to outcome assessment and follow-up exist. The 12-month follow-up duration is insufficient to capture long-term outcomes, such as the development of hip osteoarthritis, implant loosening, or late complications, potentially underestimating long-term risks. The Harris Hip Score (HHS) showed a ceiling effect at 12 months (scores >85 in both groups), reducing its sensitivity to detect subtle differences in functional recovery. Preoperative HHS values, though not statistically different, had slight numerical discrepancies that might have influenced interpretations of postoperative improvements. This suggests HHS alone is inadequate for comprehensive evaluation, and future studies should incorporate additional tools like the WOMAC or patient-reported outcome measures (PROMs). (4) Technical and procedural factors may introduce bias. The learning curve associated with the lower extremity axial bone traction device could have influenced outcomes: less experienced surgeons might have longer operation times or suboptimal reduction quality in early cases, which were not specifically analyzed. The lack of stratification by surgeon experience levels further limits the generalizability of the results. (5) The lack of randomization in treatment assignment, despite efforts to balance groups through propensity score matching, remains a critical limitation, as it hinders causal inference about the superiority of one technique over the other. Future research should address these limitations through randomized controlled designs, longer follow-up periods (>1 year), multicenter collaboration with larger samples, standardized assessment of surgeon experience, and integration of multiple outcome measures to provide more robust evidence.

## Data Availability

The original contributions presented in the study are included in the article/Supplementary Material, further inquiries can be directed to the corresponding author.
